# Modelling the interaction of structural and synaptic plasticity

**DOI:** 10.1186/1471-2202-14-S1-P416

**Published:** 2013-07-08

**Authors:** Michael Fauth, Christian Tetzlaff, Florentin Wörgötter

**Affiliations:** 1 Drittes Physikalisches Institut, Georg-Auguist Univeristät Göttingen, 37077, Germany; 2 Bernstein Center for Computational Neuroscience Göttingen, Germany

## 

Recent experiments show that learning is associated with structural changes in neural tissue [1,2]. The underlying mechanism, named structural plasticity, drives the formation of new synapses and the removal of existing ones on a timescale of days to weeks. This enlarges the potential for information storage in neuronal networks [3] and is, thus, important for understanding long-term memory formation. On shorter timescales (minutes to hours) another process - synaptic plasticity - influences the transmission efficiency (weights) of a synapse and, therefore, also contributes to information storage.

We investigate the interaction between these two processes - still widely unknown - in the following rather simple model: We use rate based neurons with the total transmission efficiency between two neurons being just the sum of weights of all synapses connecting these two neurons. Thus, the number of synapses as well as their weights influence the same quantity and we can investigate the effects arising from structural and synaptic plasticity competing on different timescales. Synaptic plasticity is modeled by Hebbian learning with weight-dependent synaptic scaling [4]. For structural plasticity we have to assume a certain number of potential synaptic sites. The formation of a synapse at each of these potential synaptic sites happens at random with a fixed formation probability. The removal of existing synapses also happens randomly, but with a probability, which depends on weight and postsynaptic activity.

Although the interaction of these processes is quite complex, we show that the system converges to a stable state. In this state the activity determines the probability distribution of number and strength of synapses between neurons. This interplay could also serve to form highly interconnected clusters, which are candidates for memory representation (cell assemblies).
